# Development of a cancer-specific survival assessment for lymph node-positive colorectal cancer patients treated with adjuvant chemotherapy

**DOI:** 10.3389/fsurg.2025.1589875

**Published:** 2025-05-12

**Authors:** Lei Zhang, Shuang Gao, Xiaoyuan Lin, Junjie Hu, Guolin Zhang, Wei Tang, Yubo Hu, Yuanpeng Wang, Liang Chu

**Affiliations:** ^1^ Department of General Surgery, The Second Affiliated Hospital of Bengbu Medical University, Bengbu, Anhui, China; ^2^ Graduate School of Bengbu Medical University, Bengbu, Anhui, China; ^3^ Center for Medical Research and Innovation, The First Hospital of Hunan University of Chinese Medicine, Changsha, Hunan, China; ^4^ Department of Radiotherapy, The Second Affiliated Hospital of Bengbu Medical University, Bengbu, Anhui, China

**Keywords:** lymph node positivity, colorectal cancer, cancer-specific survival, adjuvant chemotherapy, prognostic model

## Abstract

**Background:**

To construct a prognostic model for predicting cancer-specific survival in lymph node-positive colorectal cancer patients treated with adjuvant chemotherapy after surgery.

**Methods:**

Data were collected from the 2010–2015 SEER database and from CRC patients at the Second Affiliated Hospital of Bengbu Medical University (2017–2023). Lasso regression and random survival forest methods were used to screen ten clinicopathologic features. Cox regression analysis identified independent prognostic factors for CRC. Nomogram plot model was used to predict 1-, 3-, and 5-year survival rates, with its accuracy verified through ROC curves, calibration curves, and decision curve analysis (DCA). The X-tile software differentiated between high and low-risk groups and illustrated survival differences using Kaplan–Meier curves.

**Results:**

Age, histologic grade, stage, CEA, nerve invasion, and LNR were independent prognostic risk factors for colorectal cancer (*P* < 0.001); and LNR were the five variables used to construct the Nomogram. The area under the curve (AUC) was 0.83, 0.85, and 0.84 at 1, 3, and 5 years for the training cohort; 0.83, 0.85, and 0.84 at 1, 3, and 5 years for the internal validation cohort; and 0.83, 0.85, and 0.84 at 1, 3, and 5 years for the external validation cohort, respectively. calibration curves, C-indexes, and DCA curves validated the accuracy of the model, respectively. The survival prognosis of the high-risk group was lower than that of the low-risk group in all three data sets. (HR = 6.37, CI:6.05–6.71, *P* < 0.05; HR = 7.05, CI:6.52–7.64, *P* < 0.05; HR = 2.69, CI:1.66–4.37, *P* < 0.05)

**Conclusions:**

LNR represents a new independent prognostic factor for lymph node-positive CRC. The optimal threshold determined by the Nomogram method effectively categorizes subgroups of lymph node-positive CRC cases after surgical chemotherapy, crucial for guiding clinical treatment strategy selection.

## Introduction

Colorectal cancer (CRC) is the most prevalent malignant tumor in the gastrointestinal tract, ranking third in incidence and second in mortality worldwide (1). The primary treatments for CRC are surgery, chemotherapy and radiotherapy (2, 3). Several pathologic features have been identified that correlate with CRC prognosis, notably positive lymph nodes (pLN) as the most significant predictor (4). This method uses the absolute number of pLN for staging. However, the accuracy of this staging system remains controversial. Numerous studies have revealed a significant correlation between pLN and the total number of dissected lymph nodes (DLNs) (5). Insufficient DLNs may reduce pLN detection rates, resulting in staging bias (6).

Lymph node ratio (LNR) is defined as the ratio between positive lymph nodes (PLNs) and removed lymph nodes (RLNs) (7). Studies indicate that a lower LNR correlates with a better prognosis in breast, esophageal, gastric cancer patients (8–10). The available literature focuses on the relationship between LNR and factors such as tumor stage and lymph node metastasis (11). Preliminary studies have suggested that a high LNR is associated with a poor prognosis (12). It can also predict more extensive lymph node metastasis of tumor cells, which may influence chemotherapy efficacy and treatment strategy choices. Additionally, some reports reveal that key prognostic factors, identified as independent predictors of oncological outcomes in resected CRC, were overlooked, hindering more accurate survival prognosis for each case (13, 14). However, information on LNR's prognostic significance in CRC patients treated with adjuvant chemotherapy after surgery is still scarce. In addition, cancer-Specific Survival (CSS) measures the time from diagnosis or treatment initiation to death caused by cancer (15). Unlike Overall Survival (OS), CSS accounts only for deaths directly resulting from cancer, excluding those from other causes. CSS evaluates only cancer-caused deaths, ignoring those from other sources like heart disease, accidents, or other illnesses. This specificity makes CSS a more precise indicator of cancer treatment effectiveness (16).

Consequently, there is a need for a validated prognostic scoring system for patients with resectable colorectal cancer to facilitate personalized therapy guidance (17). In our study, we identified lymph node-positive CRC patients who underwent post-surgery chemotherapy and gathered data from the SEER database (2010–2015) and the Second Affiliated Hospital of Bengbu Medical University (2018–2023). We aimed to develop a prognostic nomogram, incorporating key factors to evaluate CSS and guide treatment for lymph node-positive patients. Additionally, we evaluated the performance of the nomogram and assessed its suitability for external validation in this study.

## Materials and methods

### Data source and patient selection

All patients were obtained from Surveillance, Epidemiology, and End Results (SEER), which includes patient-specific demographic and cancer information for the US population. The following inclusion criteria were used: (1) Diagnosis from 2010–2015; (2) Positive lymph nodes greater than 1; (3) Surgically confirmed positive lymph nodes. (4) Histological behavior: adenocarcinoma. Exclusion criteria were (1) race unknown (=8); (2) grade unknown (=241); (3) Tx (=15); (4) Nx (=4); (5) chemotherapy unknown (=814); (6) tumor size unknown (=598); and (7) M1 and Mx stage (=317).

External validation cohort data from colorectal cancer patients who received postoperative chemotherapy at the Second Affiliated Hospital of Bengbu Medical University from 2018–2023, a total of 167 cases.

### Variables identified and outcome criteria

We identified all cases from the SEER database using SEER*Stat software version 8.4.2, which is publicly available for research and does not require ethics committee approval or informed consent. Our study methodology adhered to SEER database rules and extracted information on all primary CRC patients from 2010–2015. We accessed various variables from the SEER database, including age at diagnosis, gender, site, histological grading, tumor size, T-stage, N-stage, M-stage, overall stage, CEA levels, nerve invasion, number of positive lymph nodes, number of detected lymph nodes, time, and CSS. The SEER statistical program calculates survival time in months, and the study's cut-off date was December 31, 2020. It's noteworthy that we determined the number of days in a month to be 365.24/12. The specific cause of death was colorectal cancer.

### Statistical analysis

We employed the createDataPartition function to randomly divide the research objects into development and validation queues in a 7:3 ratio, with a random seed set to 123,456. Student's *t*-test and chi-square test were used for continuous and categorical variables, respectively, to explore the baseline characteristics of patients in both groups. Categorical variables were expressed as frequencies and their proportions, and continuous variables were expressed as mean ± standard deviation (SD). In the developmental cohort, univariate Cox regression analysis was used to identify potentially important prognostic factors. They were included in multivariate Cox proportional risk regression models when their *P* values were below 0.05. All results are shown as hazard ratios (HR) and 95% confidence intervals (95% CI).

Nomogram were included for variables selected from multiple Cox models with a critical *P* value of 0.05. Nomogram were created to visualize the 3- and 5-year survival probabilities of the predicted development cohort. We assessed model performance using Harrell's consistency index (C-index) and receiver operating characteristic curve (ROC) curves and calculated area under curve (AUC). In addition, the agreement between predicted and actual outcomes for 3- and 5-year survival times was assessed by calibration plots with the rms package in Rstudio. Patients in the development cohort were categorized into three levels of risk groups based on total points gained. Also, the Kaplan–Meier method was used to analyze the differences in CSS among the three risk groups. All statistical analyses were performed using R version 4.3.1 (https://cran.rproject.org/bin/windows/base/old/3.6.3).

## Results

### Data source

We identified colorectal cancer survivors from the National Cancer Institute's Surveillance, Epidemiology, and End Results (SEER) 13-registry database by analyzing patients diagnosed between 2010 and 2015. The SEER database provides high-quality, validated data on causes of death among cancer survivors, offering valuable insights into both relative and cause-specific mortality in this population. We accessed data using SEER*Stat 8.4.1, a statistical software developed by the Surveillance Research Program of the National Cancer Institute in Bethesda, MD, which facilitates comprehensive analysis of cancer-related data. Our study was approved by the Second Affiliated Hospital of Bengbu Medical University and exempted from ethical requirements. The study was conducted in accordance with the Declaration of Helsinki (Revised 2013) to ensure compliance with ethical standards for research involving human subjects.

### Machine learning identifies clinicopathologic factors in CRC

**Figure 1:**
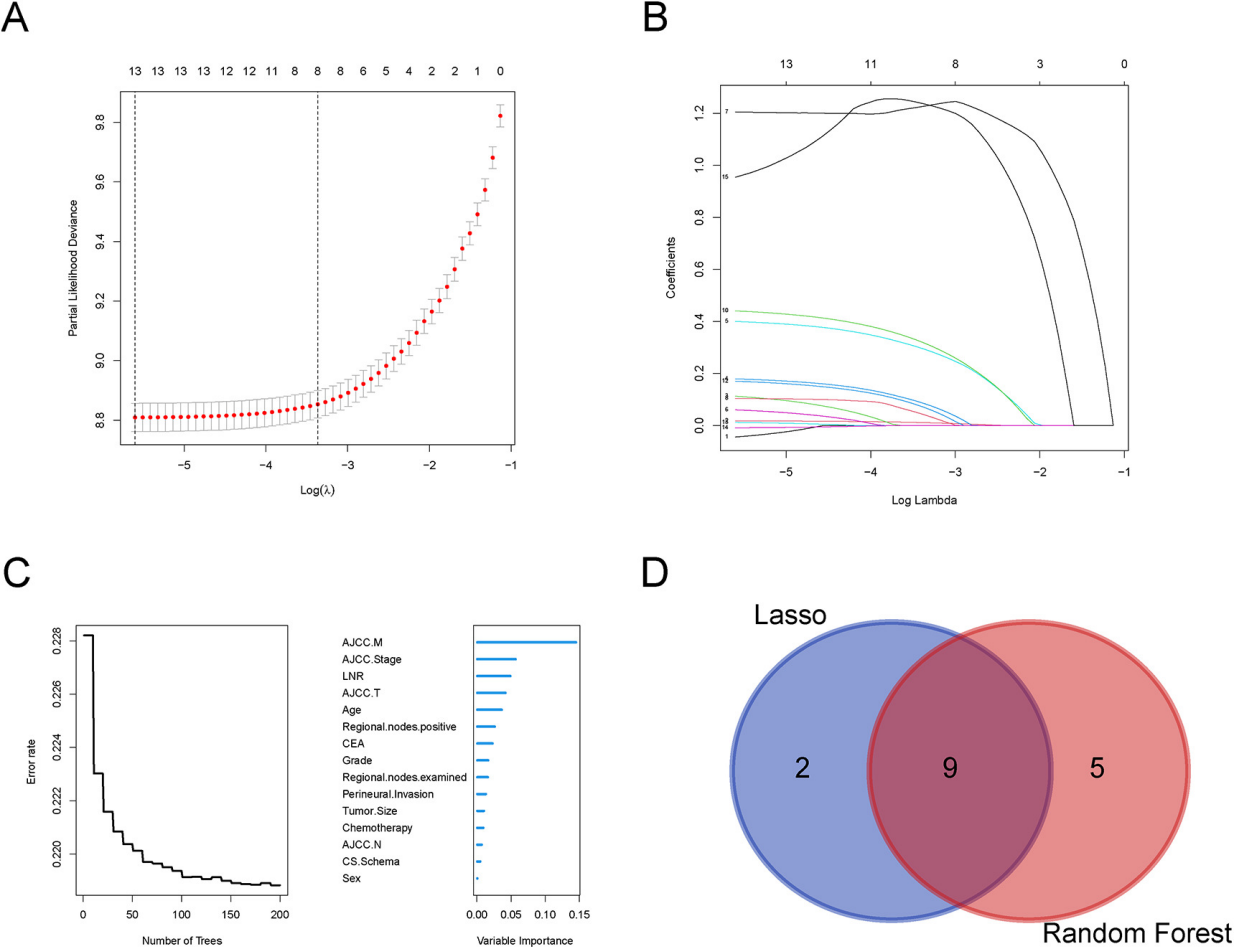
Selection of clinicopathologic factors for colorectal cancer by Lasso regression and random forests. **(A)** Partial likelihood deviance plot showing the relationship between the logarithm of the tuning parameter (*λ*) and partial likelihood deviance. **(B)** Coefficient profiles for the features across the range of log(*λ*) in the Lasso regression model; **(C)** Error rate of the Random Forest model as a function of the number of trees; **(D)** Venn diagram comparing selected variables from Lasso regression and Random Forest models.

21,705 metastatic CRC patients (2010–2015) were randomly divided into a training group (15,179) and a validation group (6,526) at a 7:3 ratio. There were no significant differences between the two groups in terms of age at diagnosis, gender, histological grade, T-stage, N-stage, M-stage, Stage, tumor size, chemotherapy, CEA, perineural invasion, number of positive lymph nodes, and number of lymph nodes detected (*P* > 0.05, Table 1), and thus the randomized grouping of the training group and the validation group was comparable. Initially, we applied lasso regression, a machine learning technique, to 13 clinicopathologic factors, identifying 11 significant factors (Figures 1A,B). Subsequently, we re-evaluated these factors using random survival forests. Factors with change weights over 0.01, including age, histological grading, tumor size, T-stage, N-stage, M-stage, overall stage, CEA, nerve invasion, and the number of positive and detected lymph nodes, were identified as primary variables (Figure 1C). By intersecting the sets from both analyses, nine key clinicopathologic factors were identified (Figure 1D). Finally, we performed univariate and multivariate Cox regression for each of the above pathologic factors, and finally determined that age, histologic grading, staging, CEA, nerve invasion, and LNR were independent prognostic risk factors for CRC (Table 2).

**Table 1:** Baseline table of colorectal cancer patients from the SEER database.

Characteristics	Total	Train	Test	*P*-value
	*N*=21,705	*N*=15,179	*N*=6,526	
Age				0.614
<60	9,833 (45.3%)	6,894 (45.4%)	2,939 (45.0%)	
>60	11,872 (54.7%)	8,285 (54.6%)	3,587 (55.0%)	
Sex				0.115
Female	10,419 (48.0%)	7,340 (48.4%)	3,079 (47.2%)	
Male	11,286 (52.0%)	7,839 (51.6%)	3,447 (52.8%)	
Grade				0.104
I	1,042 (4.80%)	705 (4.64%)	337 (5.16%)	
II	15,091 (69.5%)	10,522 (69.3%)	4,569 (70.0%)	
III	4,509 (20.8%)	3,187 (21.0%)	1,322 (20.3%)	
IV	1,063 (4.90%)	765 (5.04%)	298 (4.57%)	
AJCC.T				0.797
T1	704 (3.24%)	500 (3.29%)	204 (3.13%)	
T2	1,547 (7.13%)	1,069 (7.04%)	478 (7.32%)	
T3	13,374 (61.6%)	9,366 (61.7%)	4,008 (61.4%)	
T4	6,080 (28.0%)	4,244 (28.0%)	1,836 (28.1%)	
AJCC.N				0.670
N0	4,396 (20.3%)	3,097 (20.4%)	1,299 (19.9%)	
N1	9,996 (46.1%)	6,968 (45.9%)	3,028 (46.4%)	
N2	7,313 (33.7%)	5,114 (33.7%)	2,199 (33.7%)	
AJCC.M				0.900
M0	16,413 (75.6%)	11,474 (75.6%)	4,939 (75.7%)	
M1	5,292 (24.4%)	3,705 (24.4%)	1,587 (24.3%)	
AJCC.Stage				0.423
I	210 (0.97%)	146 (0.96%)	64 (0.98%)	
II	3,429 (15.8%)	2,437 (16.1%)	992 (15.2%)	
III	12,774 (58.9%)	8,891 (58.6%)	3,883 (59.5%)	
IV	5,292 (24.4%)	3,705 (24.4%)	1,587 (24.3%)	
Chemotherapy				0.144
No	24 (0.11%)	13 (0.09%)	11 (0.17%)	
Yes	21,681 (99.9%)	15,166 (99.9%)	6,515 (99.8%)	
CEA				0.414
Negative	11,192 (51.6%)	7,855 (51.7%)	3,337 (51.1%)	
Positive	10,513 (48.4%)	7,324 (48.3%)	3,189 (48.9%)	
Tumor Size				0.063
<=3	21,537 (99.2%)	15,050 (99.2%)	6,487 (99.4%)	
>3	168 (0.77%)	129 (0.85%)	39 (0.60%)	
Perineural Invasion				0.194
no	17,090 (78.7%)	11,988 (79.0%)	5,102 (78.2%)	
yes	4,615 (21.3%)	3,191 (21.0%)	1,424 (21.8%)	
Regional nodes positive				0.503
0	4,977 (22.9%)	3,493 (23.0%)	1,484 (22.7%)	
1–3	9,415 (43.4%)	6,572 (43.3%)	2,843 (43.6%)	
4–6	3,603 (16.6%)	2,491 (16.4%)	1,112 (17.0%)	
>=7	3,710 (17.1%)	2,623 (17.3%)	1,087 (16.7%)	
Regional nodes examined				0.535
1–11	2,323 (10.7%)	1,638 (10.8%)	685 (10.5%)	
>=12	19,382 (89.3%)	13,541 (89.2%)	5,841 (89.5%)	

**Table 2:** Univariate and multifactorial cox analysis of clinicopathologic factors in patients with colorectal cancer.

Variables	Univariate analysis		Multivariate analysis	
	HR (95% CI)	*p*-value	HR (95% CI)	*p*-value
Age	1.02 (1.01–1.02)	<0.01	1.02 (1.02–1.02)	<0.01
Grade	1.43 (1.39–1.48)	<0.01	1.2 (1.16–1.24)	<0.01
Perineural Invasion	2.04 (1.95–2.13)	<0.01	1.21 (1.15–1.27)	<0.01
CEA	2.56 (2.45–2.68)	<0.01	1.57 (1.49–1.64)	<0.01
AJCC.T	2.11 (2.04–2.19)	<0.01	1.53 (1.47–1.58)	<0.01
AJCC.N	1.85 (1.79–1.9)	<0.01	1.03 (0.98–1.08)	0.2143
AJCC.M	5.91 (5.66–6.17)	<0.01	3.42 (3.04–3.84)	<0.01
AJCC.Stage	3.97 (3.82–4.13)	<0.01	1.11 (1.01–1.22)	0.0269
LNR	10.19 (9.45–10.99)	<0.01	3.76 (3.37–4.21)	<0.01

### Nomogram construction and characterization

**Figure 2:**
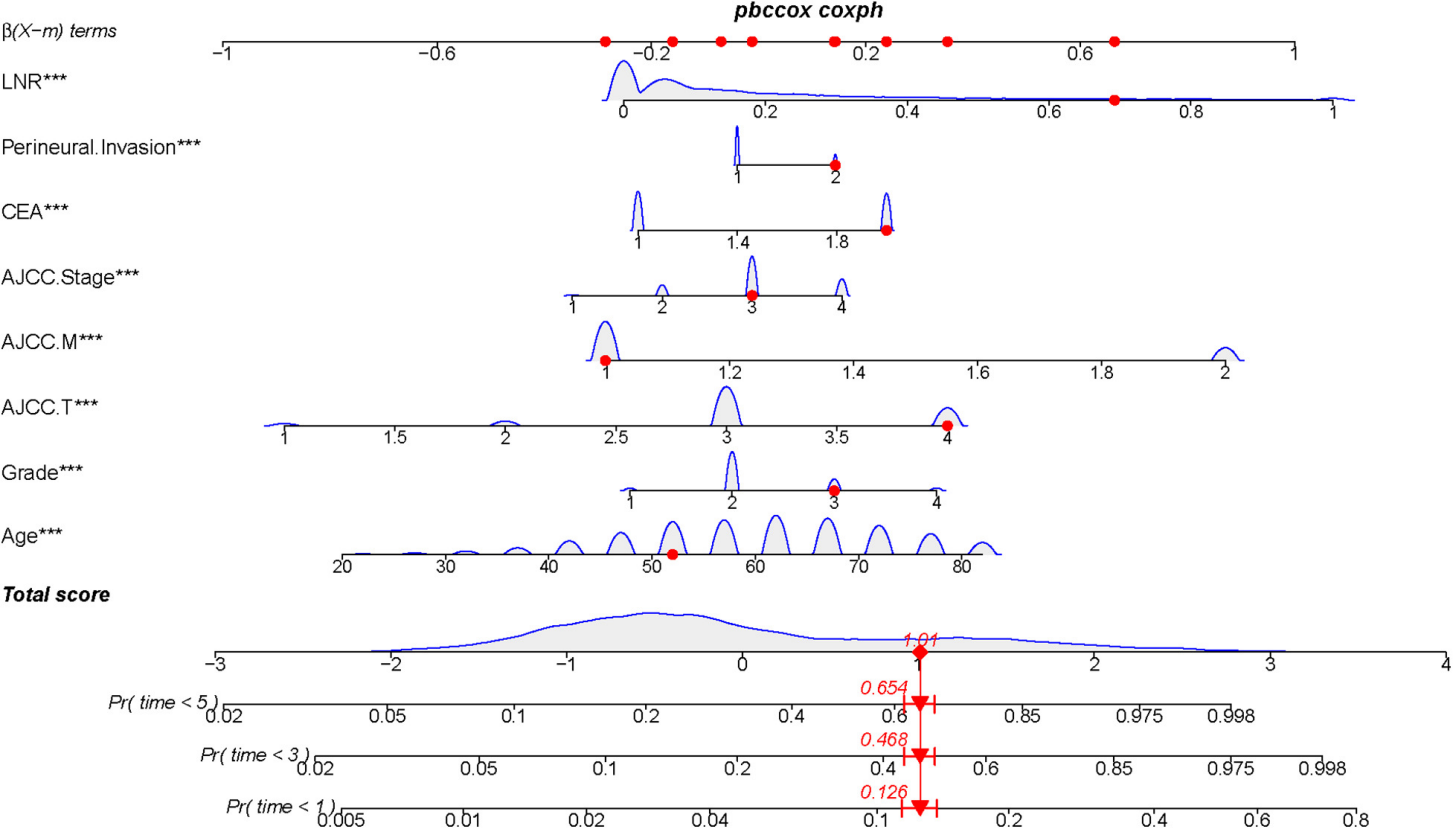
Construction of a prognostic model for colorectal cancer treated with postoperative chemotherapy by Normangram chart.

**Figure 3:**
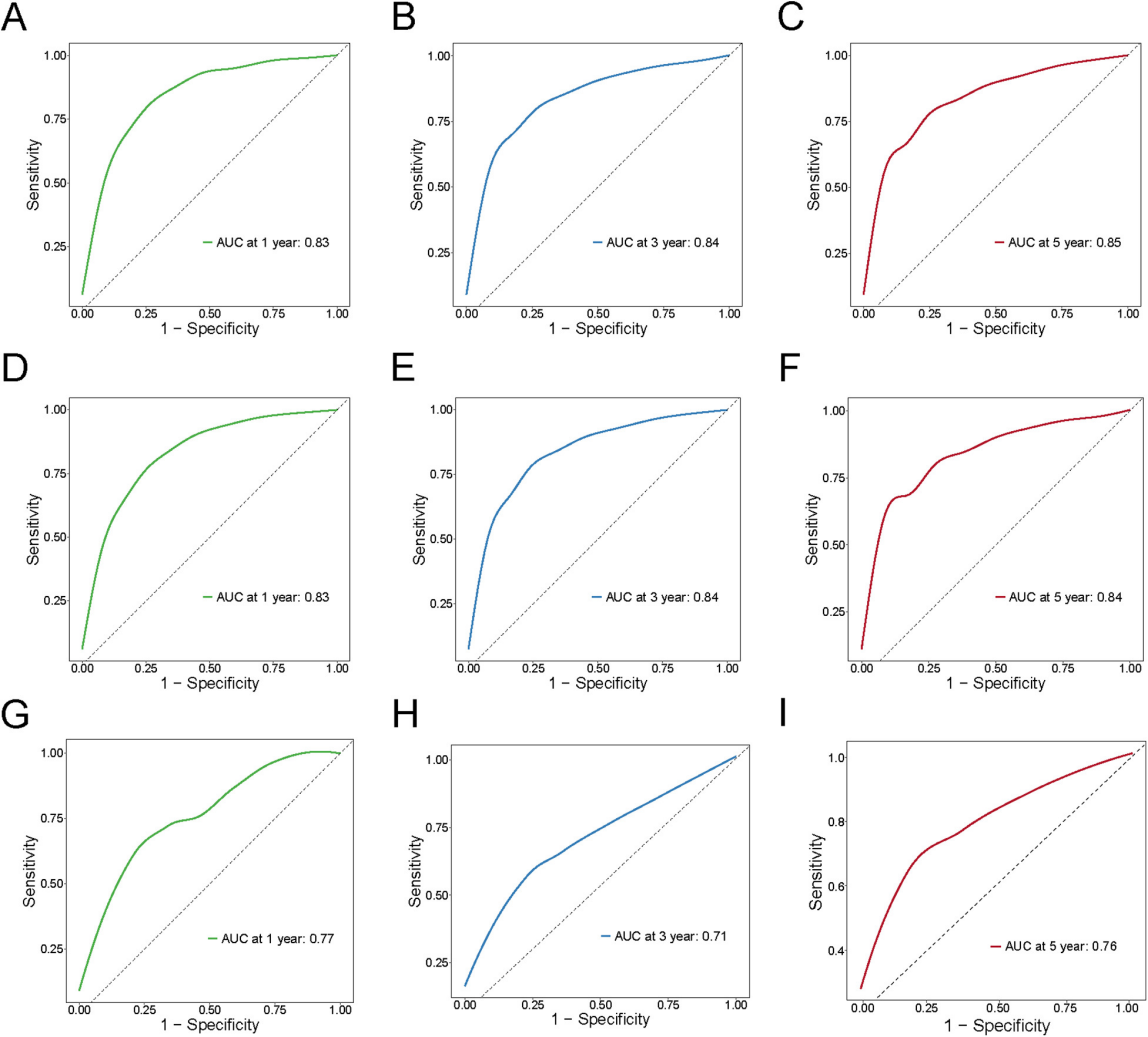
ROC curves for training cohort, internal validation cohort and external validation cohort for 1,3,5 years. **(A–C)** Training cohort; **(D–F)** Internal validation cohort; **(G–I)** External validation.

**Figure 4:**
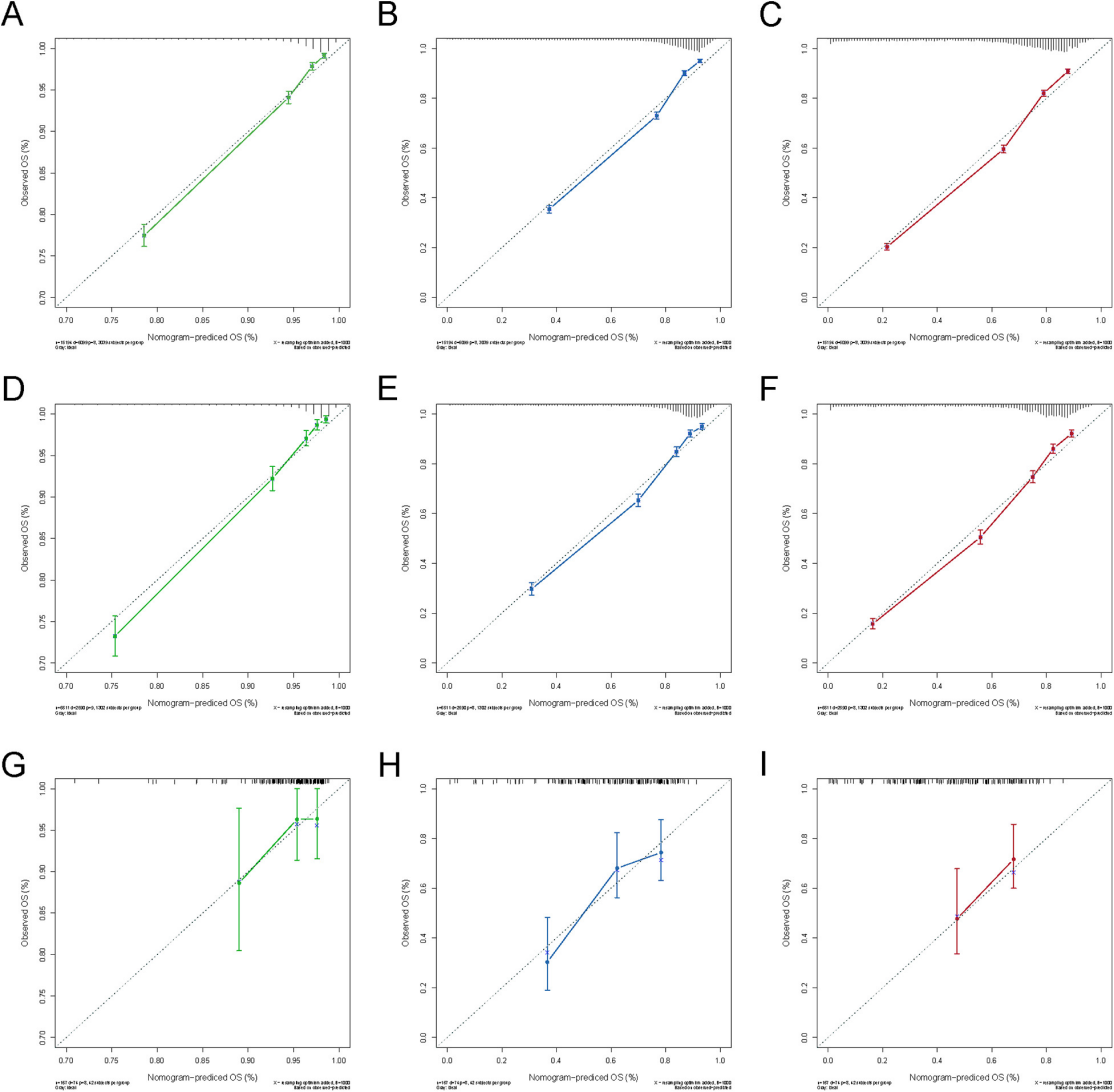
Calibration curves for 1-, 3- and 5-year OS of the training and validation cohort. **(A)** Calibration curve for 1-year OS of the training cohort; **(B)** Calibration curve for 3-year OS of the training cohort; **(C)** Calibration curve for 5-year OS of the training cohort; **(D)** Calibration curve for 1-year OS of the internal validation cohort; **(E)** Calibration curve for 3-year OS of the internal validation cohort; **(F)** Calibration curve for 5-year OS of the internal validation cohort; **(G)** Calibration curve for 1-year OS of the external validation cohort; **(E)** Calibration curve for 3-year OS of the external validation cohort; **(F)** Calibration curve for 5-year OS of the external validation cohort.

**Figure 5:**
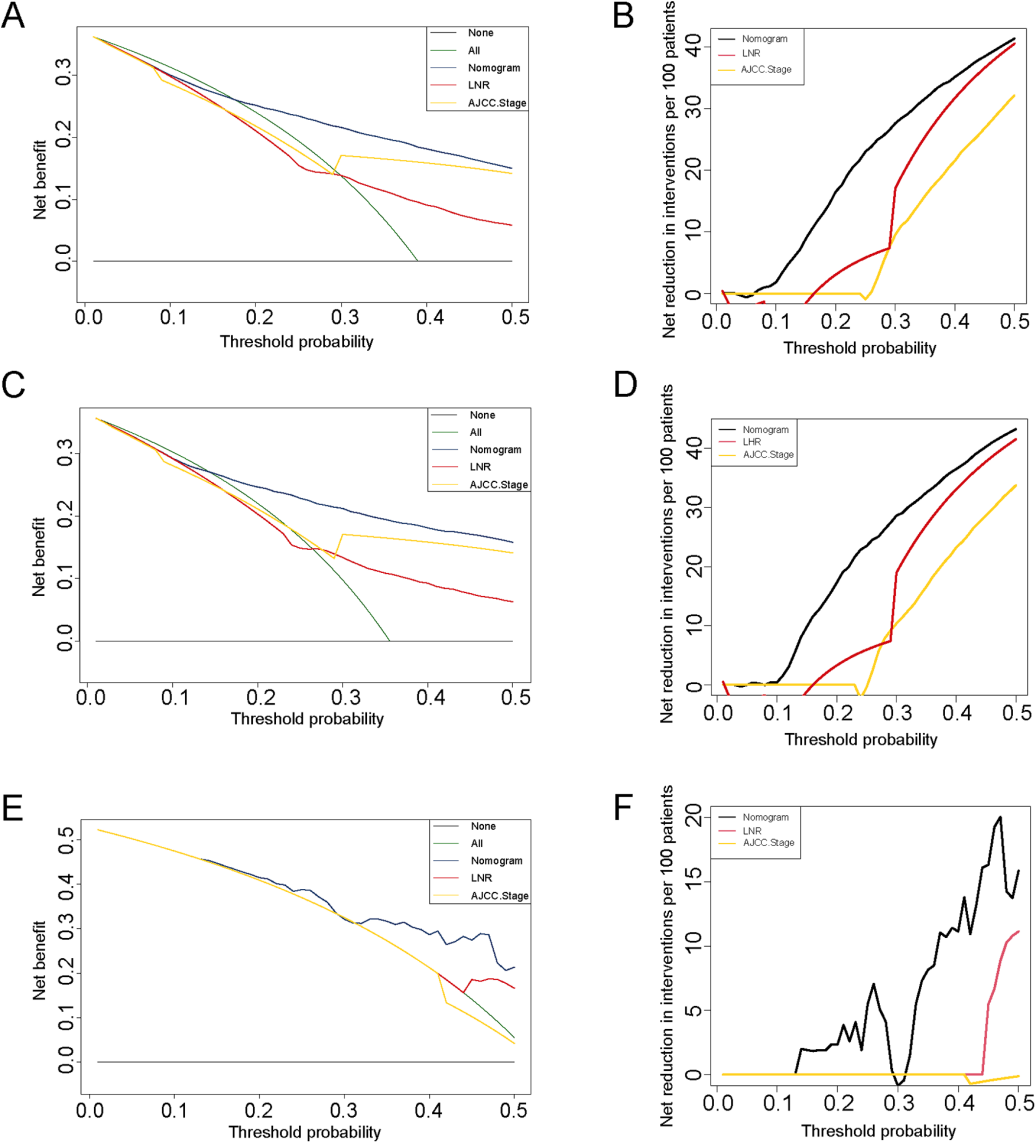
DCA curves for colorectal cancer with post-surgical chemotherapy under different strategies. **(A–B)** DCA curves and net reduction of interventions for different strategies in the training cohort; **(C–D)** DCA curves and net reduction of interventions for different strategies in the internal cohort; **(E–F)** DCA curves and net reduction of interventions for different strategies in the external cohort.

The nomogram model was constructed to predict CSS in patients using selected independent risk factors that influence their outcomes. In this model, the scores for each risk factor are summed to yield a total score, which can then be used to predict patients' CSS at 1, 3, and 5 years (Figure 2). The 1-, 3-, and 5-year areas under the curve (AUCs) for cancer-specific survival in colorectal cancer (CRC) patients were calculated using temporal receiver operating characteristic (ROC) analysis to validate the effectiveness of the nomogram in predicting CSS. The 1-, 3-, and 5-year AUCs for the training cohort were 0.84, 0.83, and 0.85 (Figures 3A–C); for the internal validation cohort, they were 0.84, 0.83, and 0.85 (Figures 3D–F); and for the external validation cohort, they were identical at 0.84, 0.83, and 0.85 (Figures 3G–I), demonstrating consistent performance across different datasets. The calibration plots illustrate that the predicted curves closely align with the ideal curves for the training cohort (Figures 4A–C), internal validation cohort (Figures 4D–F), and external validation cohort (Figures 4G–I), indicating that the CSS model demonstrates high accuracy across all datasets. Additionally, DCA curves for the training cohort,internal validation cohort, and external validation cohort, respectively, identified that the nomogram have good performance in clinical practice (Figures 5A–F). In both the training set and the test set, the C-index of the nomogram was the highest. In both the training set and the test set, the C-index of the nomogram was the highest. In the single-variable analysis, the C-index of LNR and AJCC staging demonstrated a high level of performance (Supplementary Figures S1A,B).

### The risk segmentation CRC system of CSS

**Figure 6:**
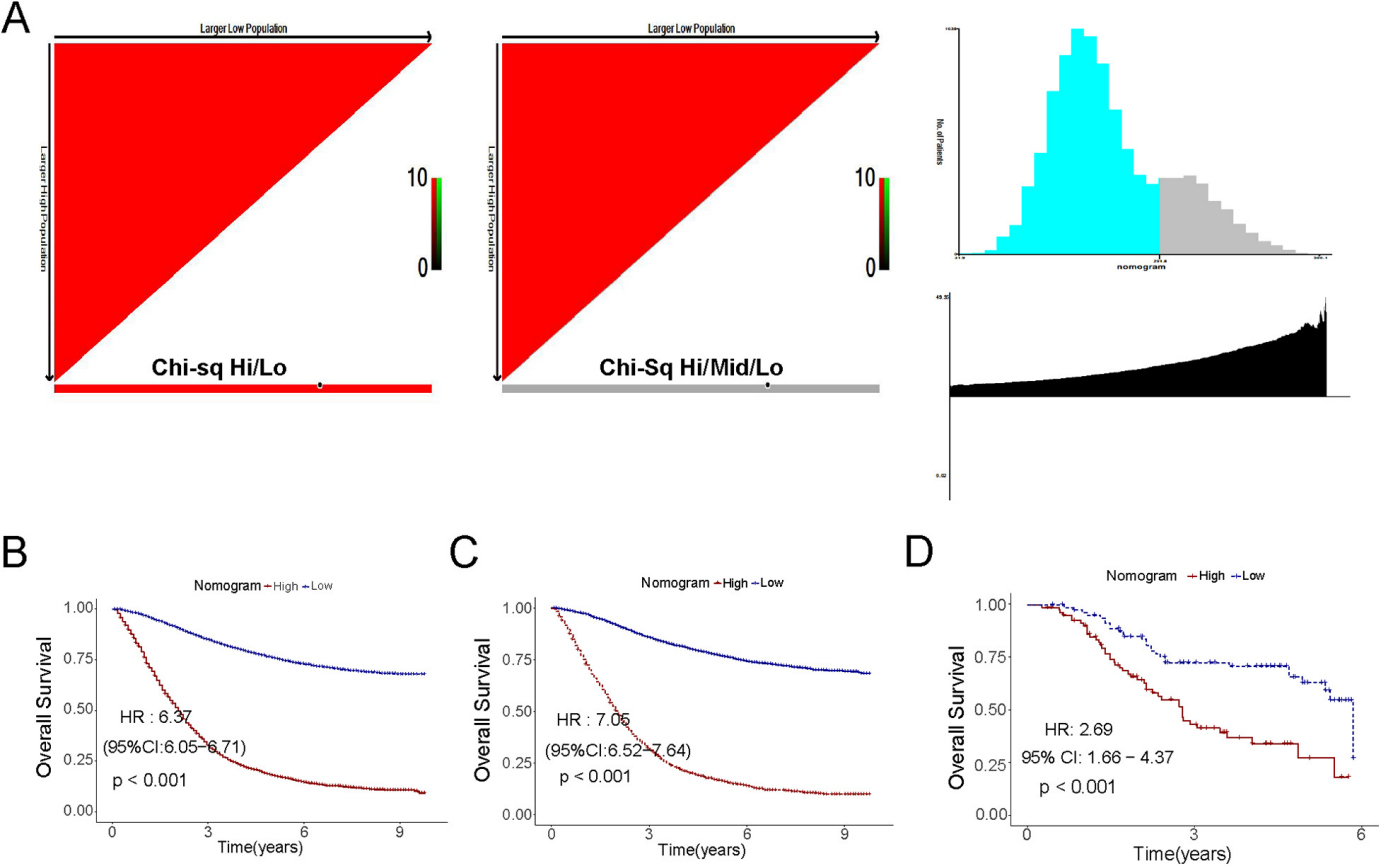
Kaplan–Meier curves of the low- and high-risk in the overall survival in the training cohort, the validation cohort and the external set. **(A)** Threshold determination by x-tile software; **(B)** The training cohort; **(C)** The internal cohort; **(D)** The external cohort.

All risk variables have been scored according to their contribution to CSS, and we used X-Tile software to select the best cutoff value of 174.53 for the scores in the training cohort (Figure 6A) (18). Based on the cutoff score, CRC patients in the training cohort were categorized into two risk subgroups: low-risk: <174.53; and high-risk: >174.53. High- and low-risk groups were classified according to the obtained scores in the internal training cohort and the external validation cohort, respectively, and a consistent difference in survival was obtained in the Kaplan–Meier curves of all three (Figures 6B–D, *P* < 0.05).

## Discussion

In recent years, the field of surgery has seen rapid and continuous technological advances. Among these, the most revolutionary breakthroughs include the Internet of Things (IoT) and robotic endoscopic technology (19). Although with the advent of gastrointestinal endoscopy, a proportion of patients were offered surgical treatment followed by standard chemotherapy (20). However, lymph node-positive colorectal cancer is considered to be a serious stage associated with high recurrence and mortality rates, and the inability of surgeons to standardize their techniques makes the number of lymph nodes detected unattainable (21). Therefore, we needed to restage CRC patients, which resulted in a relatively simple and accurate tool that incorporates only important variables related to survival outcomes, without sacrificing accuracy. Artificial intelligence and its subfields, especially deep learning, are playing an increasingly important role in various medical fields in today's world, and the diagnosis of colon cancer is also exceptional (22, 23). The accuracy of the final survival nomogram predicting outcomes far exceeded the accuracy of the individual predictors. In addition, another advantage of nomogram plots over standard multiple regression models is that they provide individual probabilities of survival at a specific point in time, rather than the concept of relative risk. Also, the use of ROC curves, C-indexes, calibration curves, and DCA curves to assess the accuracy of nomogram plots is an advantage over traditional Cox regression models. In addition, different levels of risk groups can be constructed based on the nomogram scores, and personalized counseling and follow-up arrangements can be provided to patients for different risk groups.

We compared the clinical performance of our nomogram chart with the traditional TNM classification by C index and AUC. The results showed that our model obtained higher Cindex and AUC than the TNM system in the development cohort. Macedo et al. retrospectively enrolled 1,065 patients with CRC to assess the effect of the LNR system, but did not screen patients on treatment modalities. Meanwhile, Mroczkowski, based on 7,012 lymph node-positive patients, found that the lymph node metastasis rate of colorectal cancer provided a more accurate estimate of OS (24). However, the applicability of this among all node-positive patients was limited by the exclusion of patients receiving neoadjuvant chemotherapy (25). In addition, the inclusion of molecular markers in the model did not significantly improve the performance of outcome prediction, owing to the fact that the application of molecular markers is still limited due to their unclear effect and high price (26). The nomogram in this study had a good clinical performance in predicting CSS, and the variables were relatively easy to obtain in most hospitals. Specifically, the relatively high C index and AUC at 1, 3, and 5 years in both the development and validation groups confirmed the good discriminatory ability and accuracy of the nomogram. The calibration curves also show that the predictions of the nomogram plot are in perfect agreement with the actual results.

This new nomogram for predicting the probability of CSS incorporates six factors including age, stage, histologic grade, CEA, nerve invasion, and LNR. The study demonstrated that lymph node positivity appears to be a more promising predictor of prognosis in node-positive patients than the traditional TNM system (27). Meanwhile, wang et al. found that a higher number of lymph node detections was associated with more precise lymph node staging and better survival by studying the number of lymph nodes detected in 7,694 cases of rectal cancer from the Chinese database and 21,332 cases of rectal cancer from the SEER database (28). The number of positive nodules has been shown to be strongly associated with the prognosis of patients with positive nodules (29). In addition, the patients included in our current study were CRC patients treated with adjuvant chemotherapy after surgery, which is more consistent with actual clinical treatment patterns and allows clinicians to more accurately predict the survival prognosis of CRC patients (30).

There are several strengths of this study that are worth mentioning. First, to the best of our knowledge, this is the first prognostic nomogram chart study with CSS prediction for all lymph node-positive colorectal cancer patients. Second, the relatively large number of patients in this study was sufficient to construct a well-performing prognostic nomogram plot (*n* = 11,687). Finally, the variables in the nomogram plot were readily available in most hospitals, so our nomogram plot had good applicability. Also, we categorized the study population into two risk groups based on the prognostic nomogram, making it easier to identify patients with poorer survival outcomes. This prediction was also validated in our external validation group. However, there are some limitations to this study. First, this is a retrospective study based on the SEER database, which means that the results will inevitably be affected by selection bias. In addition, we excluded patients whose variable information was unknown, which is also an important source of selection bias. Second, the SEER database has some limitations. For example, the SEER database collects information on a large number of patients from multiple regions and hospitals and does not seem to be able to balance differences in treatment and pathologic evaluation criteria. In addition, the SEER database lacks some factors that are critical for node-positive patients, such as microsatellite instability status, chemotherapeutic agents, and radiotherapy regimens (31–33). Meanwhile, novel therapies such as targeted therapies are an evolving field and more studies are needed to validate their effectiveness (34). Finally, although we added an external validation cohort to prove the validity of the model, the number of patients in the current host hospital was relatively insufficient. Therefore, it may be necessary to conduct large prospective clinical trials for validation. Therefore, we plan to initiate a multicenter prospective cohort study in the future. It is important to ensure the general applicability of our prognostic model across different patient populations, treatment modalities, and medical settings to reduce potential single-center bias.

## Conclusions

The study based on the SEER database revealed several demographics, lymph node characteristics, and therapeutic features, which were significantly associated with the cancerspecific survival of bladder cancer patients with lymph nodepositive. A prognostic nomogram was constructed and validated to predict the individualized probability of cancer-specific survival at the time of 3- and 5-year. The nomogram could contribute to patient counseling, follow-up scheduling, and selection of treatment. Nonetheless, external and prospective validation was demanded for widely applying.

## Data Availability

The datasets presented in this study can be found in online repositories. The names of the repository/repositories and accession number(s) can be found below: https://seer.cancer.gov/.

## Supplementary material

Supplementary Figure 1 Time-dependent concordance indices for prognostic factors and the nomogram. The time-dependent concordance indices of prognostic factors and the nomogram in the training and test cohorts are presented in A and B, respectively. The nomogram exhibited superior discriminative ability in both cohorts compared to individual variables, with LNR and AJCC stage showing moderate performance.
