# Supplementary figures and images for: Development of a cancer-specific survival assessment for lymph node-positive colorectal cancer patients treated with adjuvant chemotherapy
Source: Front Surg. 2025 May 12;12:1589875. doi: 10.3389/fsurg.2025.1589875 (PMC12104235; doi:10.3389/fsurg.2025.1589875)

**A**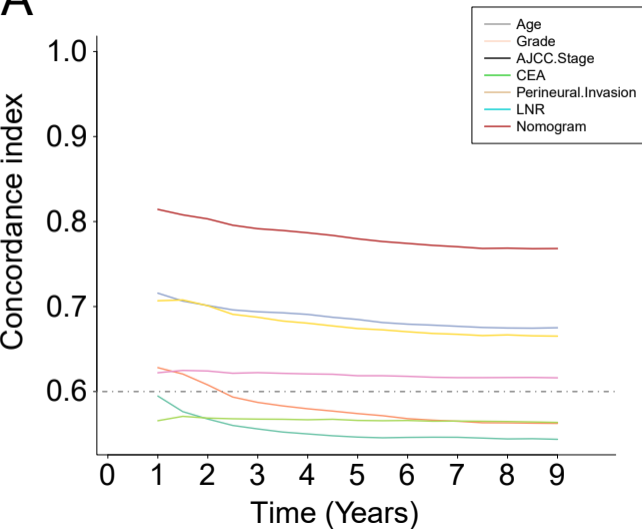**B**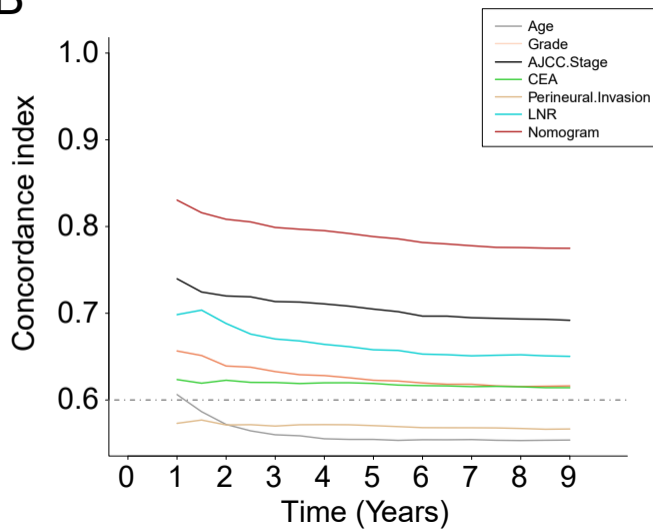

Supplement: Supplementary Figure 1 — Time-dependent concordance indices for prognostic factors and the nomogram. The time-dependent concordance indices of prognostic factors and the nomogram in the training and test cohorts are presented in A and B, respectively. The nomogram exhibited superior discriminative ability in both cohorts compared to individual variables, with LNR and AJCC stage showing moderate performance. [file Image1.pdf]
